# Multiplatform plasma metabolic and lipid fingerprinting of breast cancer: A pilot control-case study in Colombian Hispanic women

**DOI:** 10.1371/journal.pone.0190958

**Published:** 2018-02-13

**Authors:** Mónica P. Cala, Julian Aldana, Jessica Medina, Julián Sánchez, José Guio, Julien Wist, Roland J. W. Meesters

**Affiliations:** 1 Department of Chemistry, Grupo de Investigación en Química Analítica y Bioanalítica (GABIO), Universidad de los Andes, Bogotá D.C., Colombia; 2 Department of Chemistry, Universidad del Valle, Cali, Colombia; 3 Liga contra el Cáncer Seccional Bogotá, Bogotá, Colombia; Norges teknisk-naturvitenskapelige universitet, NORWAY

## Abstract

Breast cancer (BC) is a highly heterogeneous disease associated with metabolic reprogramming. The shifts in the metabolome caused by BC still lack data from Latin populations of Hispanic origin. In this pilot study, metabolomic and lipidomic approaches were performed to establish a plasma metabolic fingerprint of Colombian Hispanic women with BC. Data from ^1^H-NMR, GC-MS and LC-MS were combined and compared. Statistics showed discrimination between breast cancer and healthy subjects on all analytical platforms. The differentiating metabolites were involved in glycerolipid, glycerophospholipid, amino acid and fatty acid metabolism. This study demonstrates the usefulness of multiplatform approaches in metabolic/lipid fingerprinting studies to broaden the outlook of possible shifts in metabolism. Our findings propose relevant plasma metabolites that could contribute to a better understanding of underlying metabolic shifts driven by BC in women of Colombian Hispanic origin. Particularly, the understanding of the up-regulation of long chain fatty acyl carnitines and the down-regulation of cyclic phosphatidic acid (cPA). In addition, the mapped metabolic signatures in breast cancer were similar but not identical to those reported for non-Hispanic women, despite racial differences.

## Introduction

Breast cancer (BC) remains the most frequent type of cancer and the main cause of cancer deaths among women worldwide [1]. According to GLOBOCAN, breast cancer mortality rates in developed countries have declined in the last years, but the incidence rates continues to rise, especially in Latin America and other developing regions [1, 2]. Mortality reduction has been associated with the advances in medical diagnostic methods and the development of new therapies; however, the high heterogeneity of breast cancer still poses challenges to the understanding of its characteristic phenotype. Reported findings of breast cancer have suggested prognosis and predictive biomarkers based on alterations in genes (e.g. BRCA1 and BRCA2) [3, 4] and protein expression (e.g. mTOR, ras, PKC) [5–7]. In the past few years, metabolites have been proposed as BC markers, along with genes and proteins.

Metabolomics is a consolidated field that has enabled to observe differences in metabolic signatures generated by a pathological state such as cancer. These differences allow to postulate molecular mechanisms involved in cancer, proposing and evaluating promissory treatment targets and diagnosis tools [8–10]. Although the identification of breast cancer biomarkers by metabolomics is still at an early stage, exploratory studies have allowed highlighting alterations in aerobic glycolysis, *de novo* lipogenesis, glutaminolysis, glycerolipid, glycerophospholipid and amino acid metabolism [11–15]. These alterations have been used to identify metabolic changes associated with advanced metastatic breast cancer in cell lines [16, 17] and serum [18], as well as breast cancer subtypes in plasma [13, 19] and tissue [13, 19–21]. Moreover, the identification of suitable targets for drug development in cell lines [22–24] and therapy selection in cell lines [25] and serum [26] have also been achieved.

High-throughput analytical chemical techniques such as chromatography coupled to mass spectrometry (MS) and nuclear magnetic resonance (NMR) spectroscopy [27, 28] have been used in metabolomics, along with univariate and multivariate statistics [29, 30], in order to provide information on a large number of metabolites, in particular those with altered levels between healthy subjects and cancer patients [9, 31–34]. Metabolomics in BC has been mainly performed by NMR and MS, according to the purpose of the study and the characteristics of measured metabolites [35]. NMR has proven useful to determine significant differences in serum samples, allowing a discrimination between early and metastatic BC, regarding to amino acid, small organic molecules and general lipid content [26, 36, 37], and also to predict BC recurrence using amino acid, fatty acid and choline levels [38]. Both GC-MS and LC-MS have detected alterations that have been proposed for several biomarkers, including amino acids [38–41], small organic acids [13, 38] and fatty acids [26, 38], whereas lysophospholipid [42, 43] and carnitine [13] alterations have been found only by LC-MS. In addition, alterations in less polar lipids, such as glycerophospholipids [42, 44, 45] and glycerolipids [43, 46] have been reported by LC-MS using a lipidomics approach.

In last decades, extensive research in breast cancer has been conducted in order to understand its heterogeneity, however a comprehensive metabolic profile is still required to identify promising underlying metabolic signatures that can be used to improve breast cancer diagnosis and treatment. Besides, most studies of metabolic alterations in BC have been performed on Asian, European and North American women, little is known about the metabolic signature of BC in women from developing regions. In the present pilot study, a multiplatform metabolomic and lipidomic approach based on NMR, GC-MS and LC-MS was performed towards mapping breast cancer metabolic perturbations in Colombian Hispanic women. To our present knowledge, this is the first report of the metabolic fingerprint of BC in the Colombian population.

## Materials and methods

### Characterization of studied subjects and sample collection

Fifty-eight women between 35 and 65 years were selected for the study with the following characterization of individual groups. Control patient (CP) group: 29 healthy women with an average age of 51 ± 8 years (where ± is standard deviation) and a body mass index (BMI) with a mean value of 27 ± 3 kg/m^2^. Breast cancer patient (BCP) group: 29 women diagnosed with breast cancer, mostly invasive ductal carcinoma between stage I and III (Table 1). The average age was 50 ± 7 years and BMI mean value of 26 ± 3 kg/m^2^. All participants were non-smokers, not taking hormonal contraception, and had undergone the last dose of cancer-related treatment between three to six months before sampling, allowing at least a three month period of wash-out before sampling. The study was approved by the ethics committee of the Universidad de Los Andes and Liga contra el Cancer- Seccional Bogotá, Colombia. All participants signed the written informed consent form. Sample collection took place at Liga contra el Cancer—Seccional Bogotá, Colombia from December 2015 to January 2016. Venous blood samples were taken in the morning after overnight fasting and were collected using K_3_EDTA and Heparin Vacuette blood collection tubes for MS and NMR analysis, respectively. Once collected, the blood was centrifuged at room temperature (19°C) for 15 min at 3000 *x g*. The harvested plasma was fractionated into 100 μL aliquots in micro centrifuge tubes and stored at -80°C until analysis.

**Table 1:** Characteristics of studied subjects.

Characteristic		BCP	CP
		n = 29	n = 29
**Age Group, years (Average ± SD)**		51 ± 8	50 ± 7
**BMI, Kg/m^2^(Average ± SD)**		26 ± 3	27 ± 3
**Diagnosis**	IDC	19 (65.5%)	NA
	ILC	10 (34.5%)	NA
**Stage**	I	3 (10.3%)	NA
	II	15 (51.7%)	NA
	III	11 (37.9%)	NA
**ER status**	pos	19 (65.5%)	NA
	neg	10 (34.5%)	NA
**PR status**	pos	12 (41.4%)	NA
	neg	17 (58.6%)	NA
**HER2 status**	pos	6 (20.7%)	NA
	neg	23 (79.3%)	NA
**Surgery**	Yes	24 (82.8%)	NA
	No	5 (17.2%)	NA
**Chemotherapy**	Yes	27 (93.1%)	NA
	No	2 (6.9%)	NA
**Radiotherapy**	Yes	22 (75.9%)	NA
	No	7 (24.1%)	NA
**Hormonal Therapy**	Yes	15 (51.7%)	NA
	no	14 (48.1%)	NA

IDC: Invasive ductal carcinoma; ILC: Invasive lobular carcinoma; ER: Estrogen receptor; PR: Progesterone receptor; HER2: Human epidermal growth factor receptor 2; NA: not applicable.

### Metabolic fingerprinting by GC-MS analysis

Plasma sample preparation and metabolite analysis by GC-MS were performed as previously reported by Garcia et al. [47]. Briefly, the plasma (40 μL) was deproteinized with cold acetonitrile (1:3, −20°C), followed by a two-step derivatization: (i) methoximation with *O*-Methoxyamine hydrochloride in pyridine (15 mg/mL, room temperature, 16 h) followed by (ii) silylation with BSTFA containing 1% TMCS (70°C, 1 h). Metabolic fingerprinting (MF) was performed using an HP 6890 Series GC system equipped with an HP 6890 autosampler and an Agilent Mass Selective Detector 5973 (Agilent technologies, Palo Alto, CA, USA). Two microliters of the derivatized plasma samples were injected onto a Zebron ZB-5MSi capillary GC column (30 m x 0.25 mm x 0.25 μm) using helium as carrier gas at a constant gas flow of 1.0 mL/min. The injector temperature was set at 250°C and the split ratio to 1:10. The temperature gradient program started at 60°C held for 1 min, followed by a subsequent increase in temperature to 320°C at a rate of 10°C/min. The GC-MS transfer line, filament source and the quadrupole temperature were set at 280, 230 and 150°C, respectively. The electron ionization (EI) source was set at 70 eV and the mass spectrometer was operated in full scan mode applying a mass range from *m/z* 50 to 600 at a scan rate of 1.38 scan/s.

### Metabolic fingerprinting by LC-MS analysis

Plasma deproteinization and metabolite extraction were performed using the protocol published by Ciborowski et al. [48]. The plasma (40 μL) was mixed with a cold mixture of methanol/ethanol (1:1, −20°C) in a ratio of 1:3. MF by LC-MS was performed using an HPLC system 1200 series coupled to Q-TOF 6520 (Agilent Technologies, Santa Clara, CA, USA). Ten microliter of sample extract were injected onto a C18 column (Kinetex C18 150 mm x 2.1 mm, 2.6 μm; Phenomenex) with a guard column (Kinetex C18 20 mm x 2.1 mm, 2.6 μm; Phenomenex). LC separation was performed at 40°C using a mobile phase that consisted of 0.1% (v/v) formic acid in water (A) and 0.1% (v/v) formic acid in acetonitrile (B) at a flow rate of 0.3 mL/min. The applied gradient elution program started at 25% B increased then to 95% B in 35 min, returned to initial conditions in 1 min and was kept constant for 9 min to ensure re-equilibration of the column. Data were collected in both positive and negative electrospray ionization (ESI) modes in separate runs, using the conditions previously described [48]. During all analysis, two reference masses were continuously injected for mass correction: *m/z* 121.0509 (C_5_H_4_N_4_) and *m/z* 922.0098 (C_18_H_18_O_6_N_3_P_3_F_24_) for positive ionization mode and *m/z* 112.9856 (C_2_O_2_F_3_(NH_4_)) and *m/z* 1033.9881 (C_18_H_18_O_6_N_3_P_3_F_24_) for negative ionization mode.

### Metabolic fingerprinting by NMR analysis

Plasma samples for NMR analysis were prepared according to the procedure published by Dona et al [49]. In short, D_2_O phosphate buffer pH 7.4 (0.075M Na_2_HPO_4_·7H_2_O) with 3-trimethylsilyl propionic acid (TSP) (350 μL) was added to heparin containing plasma samples (350 μL). The mixture was centrifuged and transferred into NMR tubes. ^1^H-NMR spectra were acquired using a Bruker UltraShield 400 MHz spectrometer (Bruker Biospin, Karlsruhe, Germany). Samples were measured at 300 K employing two NMR experiments. First, a water suppression using pre-saturation pulses (zgpr, 25Hz) was carried out using the standard pulse sequence [RD—P(90°)–AQ]. Thereafter, a Carr-Purcell-Miboom-Gill (CPMG) pulse sequence was applied with a receiver gain of 90.5, a total mixing time of 78 ms (126 loops), 4 dummy scans and 64 free induction decay (FID). FIDs were multiplied by a 0.3 Hz exponential function prior to Fourier transform and only zero-order phase correction was allowed [50].

### Lipid fingerprinting by LC-MS analysis

Plasma lipids were extracted using methyl tert-butyl ether (MTBE) as previously reported by Whiley et al. [51]. In short, plasma (20 μL) was vortex-mixed with MTBE/methanol (10:2) mixture, deionized water (250 μL) was added and the upper phase containing the plasma lipid fraction was transferred into vials for LC-MS analysis. Lipid fingerprinting (LF) was performed employing the same instrumentation used for MF analysis by LC-MS. Five microliter of the lipid extract were injected onto a C8 column (Phenomenex-Luna C8 150mm x 2.0 mm, 3um). Chromatographic analysis were carried out at 60°C using a gradient elution applying 10 mM ammonium formate in Milli-Q water (A) and 10 mM ammonium formate in methanol (B) at a constant flow of 0.5 mL/min. The eluent gradient ranged from 75% to 96% B in 23 min, and was then held for 22 min at 96%B. The gradient was then increased to 100% B in 1 min and kept constant for 4 min before the gradient could return to its initial conditions in 1 min and held there for 14 min to enable column re-equilibration. Mass spectrometric detection was performed in both positive and negative ionization mode as previously described by Whiley et al. [51]. Throughout the analysis, the same reference masses were used as described in the section on MF analysis.

### Quality control samples

Quality control (QC) samples were prepared by mixing equal volumes of plasma from each BCP and CP sample. Subsequently, profiles from the QC samples were recorded following the same procedures as described above for each technique. To determine the reproducibility of plasma sample preparation and the stability of the analytical platforms used, several QC runs were performed prior to the analysis of all plasma samples until system equilibration was achieved. QC plasma samples were also analyzed after every five randomized plasma samples [52].

### Data treatment

GC-MS data treatment consisted in data deconvolution and metabolite identification using Agilent MassHunter Unknowns Analysis B.07.00, Fiehn version 2008 and NIST 14 libraries. Thereafter, retention time alignment was performed using Agilent Mass Profiler Professional B.12.1 software, and the results were exported to Agilent MassHunter Quantitative B.07.00 in order to perform the integration of each metabolite. Raw LC-MS data was treated with Agilent MassHunter Profinder Software B.06.00 using Molecular Feature Extraction (MFE) and subsequent Recursive Feature Extraction (RFE) algorithms for noise reduction, feature deconvolution and alignment. Finally, alignment and integration of the features by GC-MS and LC-MS were manually inspected and exported to Excel (Microsoft) to filter by presence and reproducibility, keeping only the metabolites detected in at least 80% of all plasma samples and a coefficient of variation (CV %) of less than 30% of the same metabolite detected in the QC samples [53].

For NMR data treatment, the spectral range was set 0.5 to 8.5, in which the spectral regions of water (4.7 to 4.9 ppm) and TSP (-0.20 to 0.20ppm) were excluded. Data were segmented and reduced by binning method with a window of 0.04 ppm and stored as a data matrix.

### Statistical analysis

Significant differences between plasma samples fingerprints from BCP and CP obtained within each technique, were evaluated by multivariate (MVA) and univariate (UVA) statistical analysis. MVA were performed using SIMCA-P+ 12.0 (Umetrics, Umea, Sweden). Unsupervised principal component analysis (PCA) was first used to evaluate the quality of the analytical system performance using the QC samples. Then, a supervised method, orthogonal partial least squares regression (OPLS-DA) was performed to maximize differences between BCP and CP, and for the selection of the variables responsible for the separation between the two different groups. Pareto scaling and logarithmic transformation were used before the statistical analysis. The accuracy of the classification was assessed by means of a double cross-validation scheme. The original data set was split into a training, test and external set before any step of statistical analysis. The number of OPLS components were chosen on the basis of a 7-fold cross-validation that was performed on the training set only, and the best model was used to predict the samples in the test set. The whole procedure was repeated 50 times with a 7 cross-validation scheme, and the results were averaged [54].

UVA was performed employing MatLab (7.10.0 Mathworks, Inc., Natick). Data normality was verified by evaluation of the Kolmogorov-Smirnov-Lillefors and Shapiro—Wilk tests and variance ratio by the Levene’s test. The *p*-value was determined by parametric (unpaired t-test) or non-parametric (Mann—Whitney U test) tests with a Benjamini—Hochberg False Discovery Rate post hoc correction (FDR).

For both LC-MS and GC-MS data, the significant variables were selected by keeping only the variables that fulfilled: 1) UVA (*p*-value <0.05 from hypothesis testing) and 2) MVA criteria (variance important in projection (VIP) with Jack-knife confident interval (JK) not including 0), while in NMR significant chemical shifts were selected only by MVA.

### Metabolite identification

Metabolites obtained by GC-MS analysis were identified using the Fiehn version 2008 and NIST 14 libraries, while significant features obtained by LC-MS were putatively identified by matching the observed accurate mass of each compound with the *m/z* values available online using following databases: METLIN (http://metlin.scripps.edu), KEGG (http://genome.jp/kegg), lipid MAPS (http://lipidMAPS.org), and HMDB (http://hmdb.ca) with the CEU Mass Mediator tool (http://ceumass.eps.uspceu.es/mediator/). Finally, some LC-MS significant metabolites were further analyzed by MS/MS analysis, in order to confirm the metabolite’s identity. For NMR, metabolites were identified by their ^1^H-NMR spectra by comparison of observed chemical shifts and signal multiplicities reported previously in the literature [55, 56].

### Pathway mapping of metabolites

Metabolic pathway analysis was performed using MetaboAnalyst 3.0 tool (http://www.metaboanalyst.ca/), which integrates two pathways analysis approaches, enrichment and topology pathway analysis. A list of compound names from identified significant metabolites was uploaded and processed using *“homo sapiens”* library [57].

## Results

Multiplatform metabolic and lipid fingerprinting analysis of BCP and CP plasma samples were conducted using four different approaches aiming at detecting the largest possible number of metabolites. The total coverage of plasma metabolites from the MS-based platforms consisted of 1428 identified metabolites, 77 by GC-MS, 298 by MF/LC-MS(+), 313 by MF/LC-MS(-), 532 by LF/LC-MS(+) and238 by LF/LC-MS(-). Furthermore, 1757 chemical shifts were detected by ^1^H NMR analysis. A comparison between the number of detected metabolites during data processing across the different analytical techniques used is presented in S1 Table, and a typical metabolic fingerprint from each platform is presented in S1–S3 Figs.

**Fig 1:**
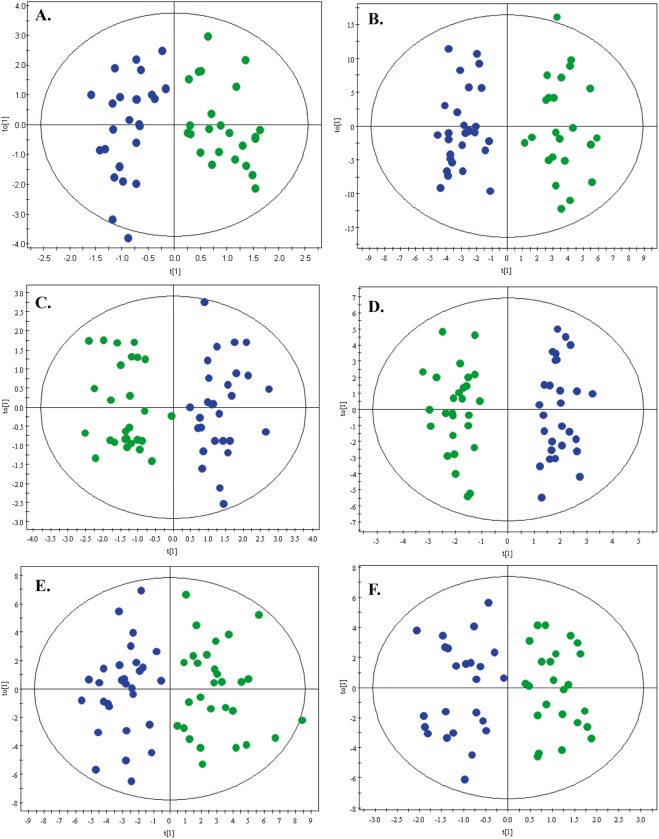
OPLS-DA models. OPLS-DA models with Log transformation and Pareto scaling for metabolic and lipid fingerprinting of breast cancer (green dots) and control (blue dots) groups. Panels: A. MF by GC-MS: *R*^*2*^ = 0.827. *Q*^*2*^ = 0.514. B. MF by NMR: *R*^*2*^ = 0.921. *Q*^*2*^ = 0.614. C. MF by LC-MS(+):*R*^*2*^ = 0.858. *Q*^*2*^ = 0.665. D. MF by LC-MS(-): *R*^*2*^ = 0.930. *Q*^*2*^ = 0.681. E. LF by LC-MS(+): *R*^*2*^ = 0.779. *Q*^*2*^ = 0.535. F. LF by LC-MS(-):*R*^*2*^ = 0.829. *Q*^*2*^ = 0.501.

The performance of the different analytical platforms was assessed by clustering of the QC samples in the PCA models (S4 Fig), assuring the acquired data quality and the conservation of biological variation over experimental bias. Using this approach, further statistical comparisons across the samples were allowed. The discrimination between BCP and CP was achieved using an OPLS-DA model for each platform, as shown in Fig 1. A clear group separation was observed in the score plots for all models, with acceptable values of predicted variance (R^2^) and predictive ability (Q^2^). Double cross-validation of the models showed that these correctly discriminated the groups correctly above 70% of classification accuracy for NMR (71%), MF/LC-MS(+) (81%), MF/LC-MS(-) (79%) and GC-MS (71%), while only above 60% for LF/LC-MS(+) (68%) and LF/LC-MS(-) (60%). Individual differentiating metabolites were determined by a combination of MVA (VIP > 1 with JK) and UVA (percentage of change > 30% and p < 0.05) criteria, obtaining 16 significant metabolites by GC-MS, 31 by MF/LC-MS(+), 50 by MF/LC-MS(-), 50 by LF/LC-MS(+) and 41 by LF/LC-MS(-) analysis.

**Fig 2:**
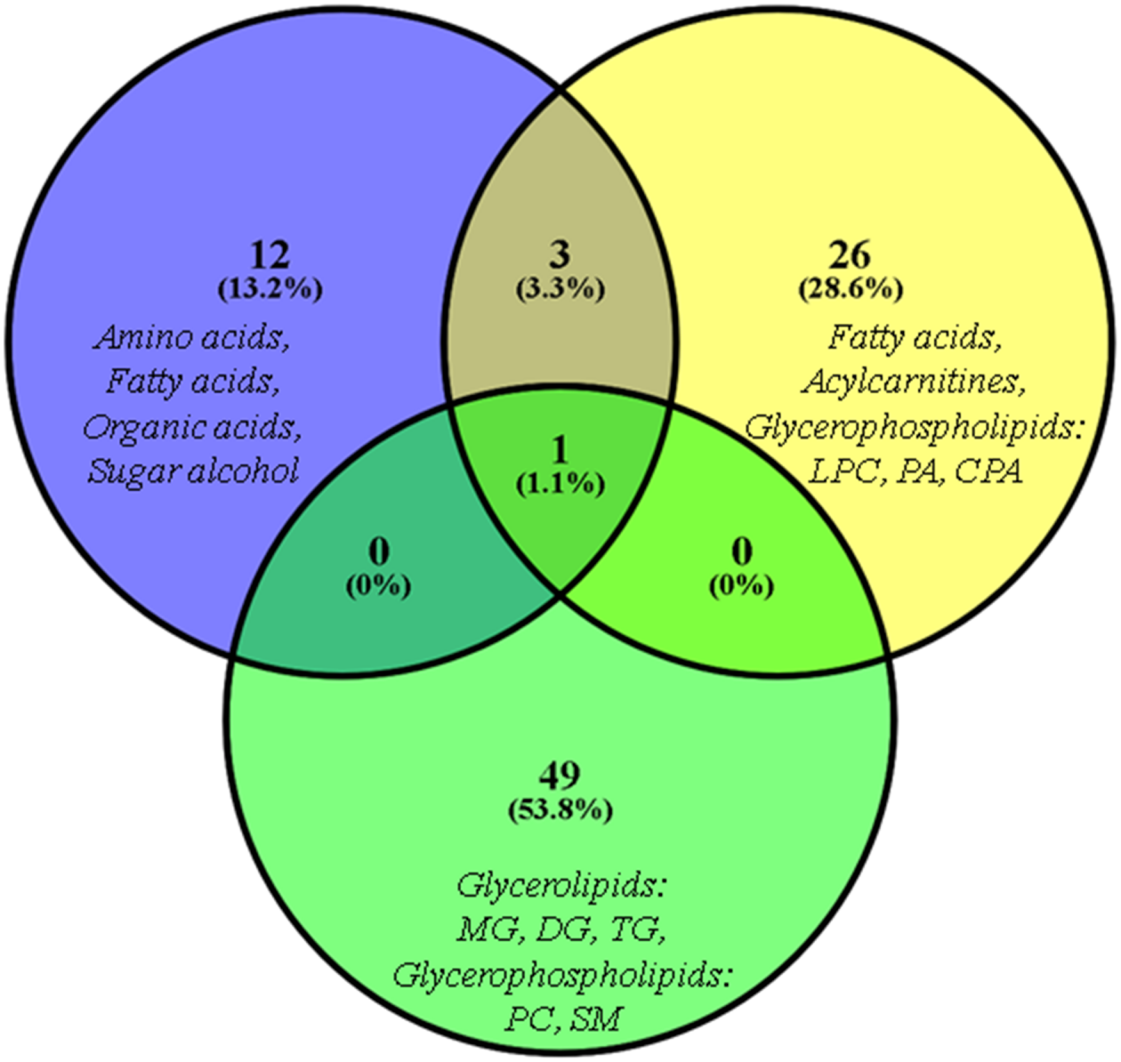
Venn diagram. Venn diagram for the number of differentiating metabolites in BCP group identified in each chromatographic technique coupled to mass spectrometry (blue circle: MF/GC-MS, yellow circle: MF/LC-MS(±), green circle: LF/LC-MS(±).

For GC-MS analysis, the significant metabolites corresponded to the chemical classes of fatty acids, organic acids and amino acids (Table 2). All identified metabolites were up-regulated in the BCP group, except for one metabolite; pyruvic acid. MF/LC-MS(±) analysis resulted in the identification of altered lipids in the plasma of BCP (Table 3), in particular, fatty acylcarnitines, fatty acids, lysophosphatidylethanolamines (LPE), lysophosphatidylcholines (LPC), phosphatidic acids (PA) and phosphatidylglycerol (PG). In addition, LF/LC-MS(±) determined significant differences in non-polar lipids such as phosphatidylcholines (PC), sphingolipids (SM) and mono, di-and triacylglycerides (MG, DG and TG) (Table 4). The number of statistical significant metabolites across MS platforms are compared in Fig 2.

**Table 2:** Compounds with statistical significance identified by GC-MS.

Compound	Molecular formula	RT (min)	Target ion	Qualifier ion (Q) *m/z*	CV for QC (%)	Change (%)	*p*-value	VIP^b^
***Amino acids and derivatives***								
Valine	C_5_H_11_NO_2_	7.146	72	55	5	+ 36	0.012	1.83
Alanine	C_3_H_7_NO_2_	7.413	116	73	6	+ 25	0.045	1.34
Isoleucine	C_6_H_13_NO_2_	8.480	86	79	7	+ 21	0.035	1.15
Serine	C_3_H_7_NO_3_	9.682	132		8	+ 110	0.019^a^	2.65
Glutamic acid	C_5_H_9_NO_4_	14.390	246	73	8	+ 27	0.047	1.26
4-Hydroxyproline	C_5_H_9_NO_3_	13.306	230	73	11	+ 55	0.042^a^	2.18
***Organic acids***								
Pyruvic acid	C_3_H_4_O_3_	6.587	174	89	6	- 22	0.014	1.35
2-Hydroxybutyric acid	C_4_H_8_O_3_	7.788	131	147	6	+ 31	0.029^a^	1.62
3-Hydroxybutyric acid	C_4_H_8_O_3_	8.350	147	73	9	+ 26	0.045	1.62
***Sugar alcohol***								
Glycerol	C_3_H_8_O_3_	9.959	205	147	4	+ 21	0.048	1.05
***Fatty acids***								
Myristic acid	C_14_H_28_O_2_	16.797	117	285	13	+ 20	0.047	1.03
Palmitoleic acid	C_16_H_30_O_2_	18.577	311	75	17	+ 60	0.017^a^	2.32
Palmitic acid	C_16_H_32_O_2_	18.757	313	117	8	+ 29	0.041^a^	1.28
Linoleic acid	C_18_H_32_O_2_	20.304	75	67	8	+ 41	0.032^a^	1.68
Oleic acid	C_18_H_34_O_2_	20.346	339	117	11	+ 39	0.021^a^	1.87
Arachidonic acid	C_20_H_32_O_2_	21.684	91	73	12	+ 43	0.021	1.41

^a^ *p* values corrected by Benjamin Hochberg (FDR correction),

^b^VIP values with Jack-Knife confidence intervals estimative not including 0, 95% confidence level, RT: retention time; CV for QC (%): CV obtained for the same feature within the set of quality control samples.; Change: percentage of change of the abundances, calculated as (Breast cancer-control)/control, the sign indicates the direction of change in BCP group.

**Table 3:** Compounds with statistical significance identified by metabolic fingerprinting using LC-MS(±).

Compound name	Molecular formula	Molecular weight (DB) g/mol	RT (min)	Mass error (ppm)	CV for QC (%)	Change (%)	*p*-value	VIP^b^	DET	CON
***Fatty acyl carnitines***										
Decanoylcarnitine	C_17_H_33_NO_4_	315.2409	11.085	0	8	+ 35	0.0091	1.75	ESI+	MS/MS
Decenoylcarnitine	C_17_H_31_NO_4_	313.2253	9.079	0	7	+ 22	0.079	1.18	ESI+	MS/MS
Dodecenoylcarnitine	C_19_H_35_NO_4_	341.2566	12.982	2	9	+ 48	0.011^a^	2.12	ESI+	MS/MS
Laurylcarnitine	C_19_H_37_NO_4_	343.2722	14.813	1	9	+ 45	0.011^a^	2.06	ESI+	MS/MS
Linoleyl carnitine	C_25_H_45_NO_4_	423.3348	20.040	1	10	+ 20	0.010	0.97	ESI+	Putative
Myristoylcarnitine	C_21_H_41_NO_4_	371.3035	18.065	0	12	+ 36	0.011^a^	1.82	ESI+	MS/MS
Oleoylcarnitine	C_25_H_47_NO_4_	425.3505	21.912	1	9	+ 29	0.0083	1.48	ESI+	Putative
Palmitoylcarnitine	C_23_H_45_NO_4_	399.3348	21.154	0	9	+ 21	0.0077	1.26	ESI+	MS/MS
Tetradecadiencarnitine	C_21_H_37_NO_4_	367.2722	14.597	2	9	+ 46	0.021^a^	2.01	ESI+	MS/MS
Tetradecenoylcarnitine	C_21_H_39_NO_4_	369.2879	16.351	0	26	+ 54	0.0044^a^	2.42	ESI+	MS/MS
***Glycerophospholipids***										
cPA(18:0)	C_21_H_41_O_6_P	420.2641	11.383	6	24	- 45	0.040^a^	1.68	ESI-	Putative
LPC(16:1)	C_24_H_48_NO_7_P	493.3168	18.548	1	11	+ 26	0.0064^a^	1.73	ESI+	MS/MS
LPC(18:1)	C_26_H_52_NO_7_P	521.3481	22.415	0	11	+ 27	0.017^a^	2.07	ESI+	MS/MS
LPC(18:4)	C_26_H_46_NO_7_P	515.3012	20.066	5	19	+ 29	0.014^a^	1.33	ESI-	Putative
LPC(20:4)	C_28_H_48_NO_8_P	557.3118	19.477	4	17	+ 24	0.033^a^	1.21	ESI-	Putative
LPE(18:0)	C_23_H_48_NO_7_P	481.3168	21.090	17	22	+ 49	0.017^b^	1.90	ESI-	Putative
LPE(18:1)	C_23_H_46_NO_7_P	479.3012	19.473	16	19	+ 29	0.015^a^	1.39	ESI-	Putative
PA(32:0)	C_35_H_69_O_8_P	648.4730	31.822	4	8	+ 35	0.00067^a^	1.74	ESI-	Putative
PA(P-31:1)	C_34_H_65_O_7_P	616.4468	28.111	8	19	+ 52	0.011^a^	1.95	ESI-	Putative
PG(22:0)	C_28_H_57_O_9_P	568.3740	10.833	1	24	+ 50	0.014	1.52	ESI-	Putative
***Fatty Acids***										
11’-Carboxy-γ-tocotrienol	C_25_H_36_O_4_	400.2614	31.615	14	24	+ 70	0.000059^a^	2.37	ESI-	Putative
9’-Carboxy-γ-tocotrienol	C_23_H_32_O_4_	372.2301	28.888	13	16	+ 41	0.0030^a^	1.74	ESI-	Putative
γ-Homolinolenic acid	C_20_H_34_O_2_	306.2550	31.203	12	25	+ 76	9.7E-07^a^	2.68	ESI-	Putative
Adrenic acid	C_22_H_36_O_2_	332.2710	31.616	17	14	+ 62	0.00074^a^	2.15	ESI-	Putative
Arachidonic acid	C_20_H_32_O_2_	304.2402	28.976	2	10	+ 45	0.014	2.28	ESI±	MS/MS
Docosapentaenoic acid	C_22_H_34_O_2_	330.2559	29.592	17	20	+ 49	0.0045^a^	1.73	ESI-	Putative
12-HETE	C_20_H_32_O_3_	320.2351	26.800	14	16	+ 35	0.017	1.35	ESI-	Putative
Linoleic acid	C_18_H_32_O_2_	280.2400	29.349	2	22	+ 50	0.00019	2.65	ESI±	MS/MS
Oleic acid	C_18_H_34_O_2_	282.2550	31.955	6	5	+ 43	0.0019^a^	2.29	ESI±	MS/MS
Palmitic acid	C_16_H_32_O_2_	256.2400	31.255	2	17	+ 34	0.0044^a^	2.04	ESI±	MS/MS

^a^ *p* values corrected by Benjamin Hochberg (FDR correction),

^b^ VIP values with Jack-Knife confidence intervals estimative not including 0, 95% confidence level, RT: retention time; CV for QC (%): CV obtained for the same feature within the set of quality control samples; Change: percentage of change of the abundances, calculated as (Breast cancer-control)/control, the sign indicates the direction of change in BCP group; DET: detection mode; CON: confirmation.

**Table 4:** Compounds with statistical significance identified by lipid fingerprinting using LC-MS(±).

Compound name	Molecular formula	Mass (Da)	RT	Mass error (ppm)	CV for QC (%)	Change (%)		*p*-value	VIP^b^	DET	CON
Oleic acid	C_18_H_36_O_2_	356.2927	5.77	2	21	+	34	0.026	1.85	ESI+	MS/MS
Stearic acid	C_18_H_36_O_2_	284.4772	7.35	0	15	+	35	0.007	2.25	ESI-	MS/MS
	***Monoacylglycerides***										
MG(18:1)	C_21_H_40_O_4_	356.2927	22.940	2	30	+	95	0.0027^a^	1.98	ESI+	Putative
MG(18:2)	C_21_H_38_O_4_	354.2770	22.520	3	27	+	80	0.000057^a^	2.24	ESI+	MS/MS
	***Diacylglycerides***										
DG(32:1)	C_35_H_66_O_5_	566.4910	21.550	3	24	+	77	0.012^a^	1.62	ESI+	Putative
DG(34:1)	C_37_H_70_O_5_	594.5223	22.980	1	25	+	77	0.0019^a^	1.85	ESI+	Putative
DG(34:2)	C_37_H_68_O_5_	592.5067	22.050	1	21	+	88	0.000049^a^	2.28	ESI+	Putative
DG(34:3)	C_37_H_66_O_5_	590.4910	21.080	0	19	+	84	0.000076^a^	2.32	ESI+	MS/MS
DG(36:2)	C_37_H_72_O_5_	620.5380	23.420	0	24	+	68	0.00035^a^	1.94	ESI+	Putative
DG(36:4)	C_39_H_68_O_5_	616.5067	17.560	2	28	+	46	0.0039^a^	1.51	ESI+	Putative
DG(38:3)	C_41_H_74_O_5_	646.5536	19.740	0	21	+	32	0.0062^a^	1.35	ESI+	Putative
DG(40:4)	C_39_H_68_O_5_	616.5067	21.600	1	22	+	75	0.00033^b^	2.09	ESI+	Putative
	***Triacylglycerides***										
TG(48:0)	C_51_H_98_O_6_	806.7363	31.700	1	10	+	41	0.00072^a^	1.56	ESI+	MS/MS
TG(48:1)	C_51_H_96_O_6_	804.7207	30.380	3	13	+	56	0.00019^a^	1.92	ESI+	MS/MS
TG(48:2)	C_51_H_96_O_6_	802.7050	31.090	1	24	+	63	0.028^a^	1.59	ESI+	MS/MS
TG(48:3)	C_51_H_92_O_6_	800.6894	29.890	1	28	+	56	0.023^a^	1.58	ESI+	MS/MS
TG(50:0)	C_53_H_102_O_6_	834.7676	33.980	3	12	+	36	0.00025^a^	1.61	ESI+	Putative
TG(50:1)	C_53_H_100_O_6_	832.7520	33.260	8	21	+	70	0.0019^a^	1.88	ESI+	Putative
TG(50:2)	C_53_H_98_O_6_	830.7363	31.700	15	18	+	87	0.0013^a^	2.27	ESI+	Putative
TG(50:3)	C_53_H_96_O_6_	828.7207	31.690	1	17	+	89	0.00015^a^	2.28	ESI+	MS/MS
TG(50:4)	C_53_H_94_O_6_	844.7389	30.380	3	27	+	99	0.000053^a^	2.43	ESI+	MS/MS
TG(52:0)	C_55_H_106_O_6_	862.7989	37.050	2	26	+	71	0.00083^a^	1.94	ESI+	MS/MS
TG(52:1)	C_55_H_104_O_6_	860.7833	34.890	5	16	+	52	0.00027^a^	1.85	ESI+	MS/MS
TG(52:2)	C_55_H_102_O_6_	858.7676	34.000	10	14	+	45	0.00019^a^	1.80	ESI+	MS/MS
TG(52:3)	C_55_H_100_O_6_	856.7520	32.360	9	15	+	66	0.000071^a^	2.05	ESI+	MS/MS
TG(52:4)	C_55_H_98_O_6_	854.7363	32.360	1	14	+	66	0.00019^a^	2.02	ESI+	MS/MS
TG(52:5)	C_55_H_96_O_6_	852.7207	30.990	0	21	+	79	0.0012^a^	2.17	ESI+	MS/MS
TG(54:2)	C_57_H_106_O_6_	886.7989	36.840	9	18	+	64	0.000076^a^	2.08	ESI+	MS/MS
TG(54:3)	C_57_H_104_O_6_	884.7833	37.070	1	30	+	90	0.00077^a^	2.13	ESI+	MS/MS
TG(54:4)	C_57_H_102_O_6_	882.7676	34.900	1	19	+	63	0.00022^a^	2.02	ESI+	MS/MS
TG(54:5)	C_57_H_100_O_6_	880.7520	33.890	1	20	+	69	0.00053^a^	1.97	ESI+	Putative
TG(54:6)	C_57_H_98_O_6_	878.7363	31.590	2	10	+	77	0.00063^a^	2.08	ESI+	MS/MS
TG(56:5)	C_59_H_104_O_6_	908.7833	36.100	0	16	+	77	0.0053^a^	2.17	ESI+	MS/MS
TG(56:6)	C_59_H_102_O_6_	906.7676	34.790	0	23	+	96	0.000014^a^	2.60	ESI+	MS/MS
TG(56:7)	C_59_H_100_O_6_	904.7520	33.300	0	19	+	87	0.0000093^a^	2.39	ESI+	MS/MS
TG(56:8)	C_59_H_98_O_6_	902.7363	31.730	3	16	+	115	0.0000052^a^	2.65	ESI+	MS/MS
TG(58:9)	C_61_H_100_O_6_	928.7520	32.450	0	9	+	72	0.0000093^a^	2.22	ESI+	Putative
	***Phosphatidylcholines***										
PC(P-31:1)	C_39_H_76_NO_7_P	701.5359	20.740	4	16	+	33	0.0036^a^	1.35	ESI+	MS/MS
PC(32:1)	C_40_H_78_NO_8_P	731.5465	20.680	0	18	+	40	0.0027^a^	1.49	ESI+	MS/MS
PC(36:0)	C_44_H_88_NO_8_P	789.6248	21.810	6	23	+	44	0.0019^a^	1.60	ESI+	MS/MS
PC(36:1)	C_44_H_86_NO_8_P	787.6091	21.790	0	23	+	49	0.0018^a^	1.68	ESI+	MS/MS
PC(O-36:4)	C_44_H_82_NO_7_P	765.5672	21.330	1	21	+	40	0.000058^a^	1.76	ESI+	MS/MS
PC(38:4)	C_46_H_84_NO_8_P	809.5935	21.810	10	23	+	41	0.0021^a^	1.54	ESI+	MS/MS
PC(P-38:4)	C_46_H_84_NO_7_P	793.5985	22.130	2	28	+	34	0.0066^a^	1.36	ESI+	MS/MS
PC(38:6)	C_46_H_80_NO_8_P	805.5622	19.520	0	13	+	36	0.0000014^a^	1.68	ESI+	MS/MS
PC(38:7)	C_46_H_78_NO_8_P	803.5465	19.540	0	13	+	35	0.00016^a^	1.67	ESI+	MS/MS
PC(40:5)	C_48_H_86_NO_8_P	835.6091	21.980	0	23	+	43	0.00017^a^	1.65	ESI+	MS/MS
PC(40:6)	C_48_H_84_NO_8_P	833.5935	21.120	0	18	+	43	0.000014^a^	1.80	ESI+	MS/MS
	***Sphingolipid***										
SM(d41:2)	C_46_H_91_N_2_O_6_P	798.6615	22.740	1	26	+	34	0.0099^a^	1.29	ESI+	MS/MS
SM(d42:2)	C_47_H_93_N_2_O_6_P	812.6771	23.340	0	27	+	32	0.014^a^	1.15	ESI+	MS/MS

^a^ *p* values corrected by Benjamin Hochberg (FDR correction),

^b^VIP values with Jack-Knife confidence intervals estimative not including 0, 95% confidence level, RT: retention time; CV for QC (%): CV obtained for the same feature within the set of quality control samples; Change: percentage of change of the abundances, calculated as (Breast cancer-control)/control, the sign indicates the direction of change in BCP group; DET: detection mode; CON: confirmation.

For ^1^H-NMR analysis, a total of 519 chemical shifts in the spectral range were found statistically significant for group differentiation. These chemical shifts correspond to the region of lipids, lactate and the amino acid valine (Table 5). These regions were integrated and evaluated by UVA, and the data allows to conclude that lactate, valine and lipids were statistically significant with a *p*-value < 0.05.

**Table 5:** Chemical shifts of compounds with statistical significance identified by ^1^H-NMR.

Metabolite	Assignment ^1^H (δ)	Multiplicity	Trend	*p*-value	VIP
Valine	1.02	d	↑	0.019^a^	1.41
	1.07	d	↑	0.022^a^	1.95
	2.31	m	↑	0.031^a^	1.96
Alanine	1.49	d	↑	0.025^a^	2.18
Lactate	1.33	d	↑	0.0014^a^	2.88
	4.11	q	↑	0.0040^a^	1.18
Lipids	0.8	m	↑	0.014^a^	2.25
	1.28	m	↑	0.014 ^a^	1.27
	2.04	m	↑	-	1.16
	5.26–5.33	m	↑	-	1.64

^a^ *p* values corrected by Benjamin Hochberg (FDR correction), Trend: Regulation in BCP group: ↑ Up regulated, ↓ Down regulated, VIP: variance important in projection from OPLS-DA model with Jack-Knife confidence intervals estimative not including 0, 95% confidence level.

## Discussion

Our results show that a multiplatform approach for metabolic and lipid fingerprinting allows a wide coverage of different metabolite classes in plasma. The combination of these approaches enabled the detection of 1450 metabolites in total, of which 95 were significantly altered in BCP, including amino acids (7), organic acids (3), sugar alcohols (1), fatty acyls (15), fatty acylcarnitines (10), glycerophospholipids (24) and glycerolipids (35). Fig 2 compares the number of altered metabolites identified by MS-based platforms, where 13%, 27% and 49% of the alterations were determined exclusively by GC, MF/LC(±) and LF/LC(±), respectively. Only oleic acid was common on all platforms.

**Fig 3:**
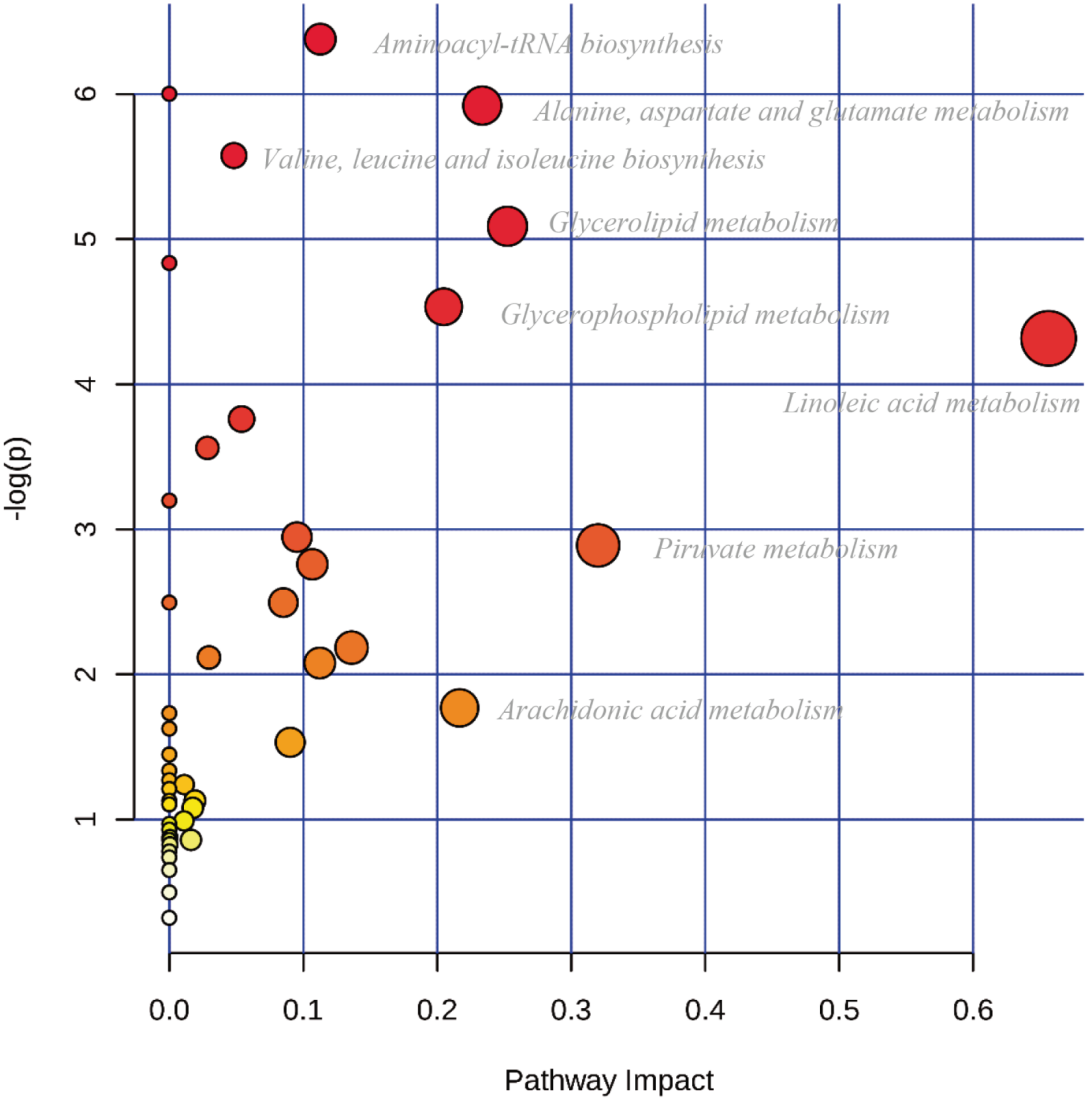
Pathway analysis. Pathway analysis displaying metabolic pathways arranged by scores from pathway enrichment (y axis) and from topology analysis (x axis) using MetaboAnalyst 3.0 tool. The color and size of each circle is based on *p*-values and pathway impact values, respectively [57].

The pathway analysis shows perturbations in the biosynthesis of aminoacyl-tRNA and several amino acids, as well as in the metabolism of fatty acids, glycerolipids and glycerophospholipids (Fig 3 and S3 Table). GC-MS and NMR both identify shifts in the glycolytic pathway [14]. The observed down-regulation of pyruvic acid and the up-regulation of lactic acid and alanine (Tables 1 and 4) are in accordance with the Warburg effect [58]. In cancer cells, energy metabolism shifts occur in order to generate energy, mainly by pyruvate to lactate conversion, regardless of oxygen concentration [59]. This generates a decrease in pyruvate and an increase in lactate. The depletion of pyruvate also affects the tricarboxylic acid (TCA) cycle. Cancer cells promote the conversion of glutamine to glutamic acid via glutaminolysis in order to maintain the TCA cycle [12, 60–62] and to provide amino groups for serine biosynthesis. Both glutamic acid and serine were detected as significantly upregulated (Table 2).

All three analytical platforms were consistent in indicating an overall upregulation of fatty acyls (FA) (Tables 1–4). This is expected considering that FA can be used as signalling molecules and an energy source in themselves, and also as building blocks for the synthesis of complex lipids. For that reason, FA are crucial to maintaining cancer cell proliferation, migration, survival and tissue invasion [13]. In breast cancer, a high demand of FA from *de novo* synthesis has been reported [63] as a response to the overexpression of several enzymes, such as fatty acid synthase (FASN) [23, 64], acetyl-CoA carboxylase (ACC) and ATP citrate lyase (ACLY) [65]. The differentiating FA included palmitic (16:0), oleic (18:1), linoleic (18:2) and arachidonic acids (20:4), as well as 12-HETE, which have been proposed as potential biomarkers in breast cancer [62, 66]. For instance, linoleic acid can modulate BRCA1 gene expression and increase 12-HETE and 15-HETE production in BC [67], thereby promoting proliferation, angiogenesis and immunomodulation in tumors [68].

**Fig 4:**
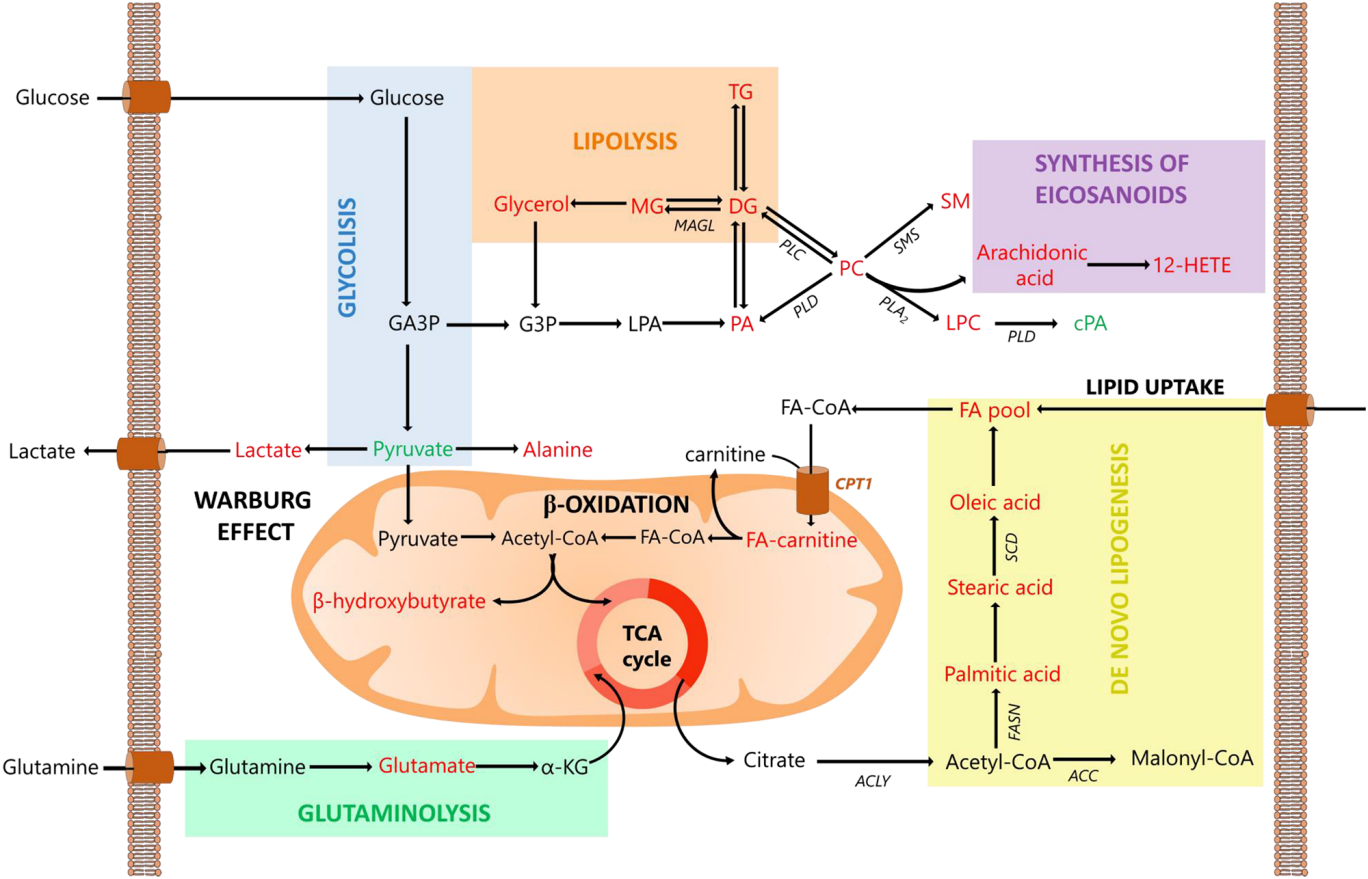
Significant altered pathways in breast cancer. Significant altered pathways in breast cancer according to multiplatform findings in blood plasma. In red up-regulated and in green down-regulated metabolites. Abbreviations: 12-HETE, 12-hydroxyeicosatrienoic acid; α-KG, α-ketoglutarate; ACC, acetyl-CoA carboxylase; ACLY, acetyl-CoA lyase; cPA, cyclic phosphatidic acid; CPT1, carnitine palmitoyltransferase I; DG, diacylglycerides; FA, fatty acid; FA-CoA, fatty acyl-CoA; FA-carnitine, fatty acyl-carnitine; FASN, fatty acid synthase; GA3P, glyceraldehyde 3-phosphate; G3P, glycerol 3-phosphate; LPA, lysophosphatidic acid; LPC, lysophosphatidylcholine; MAGL, monoacylglycerol lipase; MG, monoacyl glycerides; PA, phosphatidic acid; PC, phosphatidylcholine; PLA2, phospholipase A2; PLC, phospholipase C; PLD, phospholipase D; SCD, stearoyl-CoA desaturase; SM, sphingomyelin; SMS, sphingomyelin synthase; TCA cycle, tricarboxylic acid cycle; TG, triacylglycerides.

FA can also be consumed through β-oxidation, producing key substituents to providing the energy needed for cancer cell survival [69]. For this purpose, carnitines are used as shuttle system to transport long chain FA inside the mitochondria. Carnitine palmitoyltransferase 1 (CPT1), which catalyzes the transfer of the fatty acid moiety from acyl-coenzyme A (CoA) to a long-chain acylcarnitine, has been reported as overexpressed in breast cancer [70, 71]. Moreover, increased levels of several carnitines in BC were reported by Shen et al., including hexanoylcarnitine, octanoylcarnitine and *cis*-4-decenoylcarnitine [13]. Therefore, the up-regulation of (16:0), (18:1) and (18:2) fatty acyl carnitines shown in Table 3, suggest transport of *de novo* synthetized FA to mitochondria, where they are used to energy production via β-oxidation. This is also supported by the up-regulation of the 3-hydroxybutyric acid (a ketone body), observed by GC-MS. An overview of the basic relationships between the pathways described is presented in Fig 4.

A comprehensive understanding of the perturbations in glycerophospolipid metabolism was established both by metabolic and lipid approaches based on LC-MS. While LF/LC-MS(±) analysis found alterations in phosphatidylcholines (PC), MF/LC-MS(±) analysis found changes in phosphatidic acid (PA), lysophosphatidylcholines (LPC) and lysophosphatidylethanolamines (LPE). PC are the most common glycerophospholipids because of their structural roles and since they serve as a precursor of signalling molecules such as arachidonic acid, diacylglycerol (DG) and PA [72–74]. The relevance of PC has been examined in several studies which have determined alterations in BC samples [75–77]. As shown in Table 4, all differentiating PC species were found up-regulated in the BCP group, including some already reported as PC(32:1) [78–81], PC(36:0) [45], PC(36:1) [78–80], PC(38:4) [78] [79], PC(40:5) [17], and PC(40:6) [17, 79]. It has been suggested that PC accumulation in BC is caused by an unbalance between a high activity of the enzyme choline kinase in the anabolic pathway [73, 82, 83] and the activity of the enzymes phospholipases A_2_ (PLA_2_), phospholipase C (PLC) and phospholipase D (PLD) in the catabolic pathway [73, 84–87].

Lysophospholipids can be produced by PLA_2_ activity over PC and others glycerophospholipids. Lyso-type lipids have specific actions in cancer cells, such as activation of specific G-protein coupled receptors by LPC [88] or the increase of intracellular Ca^2+^ by LPE [89]. Therefore, the observed up-regulation of LPC and LPE species are suggested to play a role in cancer cell signaling, in agreement with previous published studies that found alterations in LPC (16:1), LPC (18:1) [17, 90, 91], LPC (20:4) [45, 92], LPE (18:0) [91], LPE (18:1) [91]. PC cleavage can also be performed by PLD to generate PA, such as the PA (32:0) and PA(P-31:1) found in this study. These acids can act as messengers that directly binds to the mammalian target of rapamycin (mTOR) to activate this anti-apoptotic pathway [93].

Nevertheless, a structural analogue of lysophosphatidic acid (LPA), the cyclic phosphatidic acid cPA (18:0) was found to be down-regulated (Table 3), which is consistent with its activity to inhibit cell proliferation, platelet aggregation, and metastasis in cancer [94]. To our present knowledge, it is the first report of cPA alterations in breast cancer plasma samples.

Finally, lipid fingerprinting by LC-MS(±) was the only analytical platform able to detect glycerolipids as differential metabolites (S2 Table). As shown in Table 4, several MG, DG and TG species were found up-regulated in the BCP group, which is consistent with previous reports in breast cancer [46, 91, 95–97]. MG can be used as a source of FA in cancer cells in order to maintain an FA pool always available, given the overexpression of monoacylglycerol lipase (MAGL) as reported by Nomura et al [98]. DG are important intermediates of the lipid metabolism and cellular signalling. Several studies have reported alterations in DG concentrations in diseases like breast cancer [46] and various other cancer types [99], by affecting the protein kinase C (PKC) [86, 100, 101]. TG are central to energy storage and as a source of building blocks for complex lipids; therefore, high TG levels have been associated with breast cancer progression and aggressiveness [102–106]. Raised TG production has been suggested as a cell strategy to decrease the cytotoxicity generated by the high amount of free FA in the cytoplasm [107, 108]. Although TG upregulation also could be related to the dietary fat, a direct link to BC has not been proven [95, 96].

Previous studies on BC in Colombian women of Hispanic origin have been focused on genomics. These studies have revealed several disparities in the gene mutations spectra when compared to other Hispanic families in United States [109] or other Central/South America countries [110, 111]. Considering that genotypic differences can be related with phenotypic ones, the present study provides an insight into the alterations of plasma metabolites in Colombian women, which is helpful for further personalized and population-based medicine approaches. A comprehensive view of all the alterations found in this multiplatform study are in accordance with the general plasma metabolic signatures associated with BC for other populations, including the glycolytic pathway, amino acid and lipid metabolism. However, some of the differentiating metabolites have not yet been reported for BC, such as cPA and several FAcyl-carnitines, which might be reflecting genotype and environmental disparities of BC in Latin woman of Hispanic origin. Although this pilot study allowed the exploration of metabolic perturbations in Colombian Hispanic women with BC, it will be important to consider for further targeted studies and/or biomarker validation a much larger cohort of women with BC in early stage, without any previously treatment.

## Conclusion

Through metabolic and lipid fingerprinting, a comprehensive characterization of metabolite alterations of breast cancer in Colombian Hispanic women was achieved. This multiplatform study demonstrated the complementarity of the different analytical technologies in non-target approaches, allowing to observe modifications in a wide range of metabolite levels. Altered metabolites belonging to glutaminolysis, amino acids, fatty acids, glycerolipid and glycerophospholipid metabolism were observed. Most of the alterations found agreed with the metabolic signatures reported previously for BC; nevertheless, in this study, new metabolites were observed that had not yet been reported, such as the down-regulation of cyclic phosphatidic acid cPA (18:0) or the up-regulation of several long fatty acylcarnitines. These findings not only provided a map of metabolic perturbations in BC, but also demonstrated that the metabolic signature of BC in Hispanic women is comparable to the metabolic signature reported for Asian, European and North American women, regardless of the large variations in this heterogeneous disease. Determining metabolic disturbances in specific populations may be promising for patient stratification, regarding the selection of an appropriate therapy or the development of new therapeutic targets.

## Supporting information

S1 Fig Comparison of GC-MS chromatograms. Comparison of GC-MS chromatograms (truncated at 30 minutes) for breast cancer (green) and control (blue). Identified metabolites: 1. *N*-Ethylglycine I. 2. Pyruvic acid. 3. Lactic acid. 4. Glycolic acid. 5. Valine I. 6. Alanine I. 7. 2-Ketoisocaproic acid I. 8. Acetoacetate I. 9. Glycine I. 10. 2-Hydroxybutyric acid I. 11. Sarcosine. 12. Acetoacetate II. 13. Oxalic acid. 14. *p*-Cresol. 15. Leucine I. 16. 3-Hydroxybutyric acid. 17. 2-etoisocaproic acid I. 18. N-methylalanine. 19. Proline I. 20. Isoleucine I. 21. 2-Ketoisocaproic acid II. 22. Malonic acid I. 23. Valine II. 24. Glyceraldehyde II. 25. Benzoic acid. 26. Urea. 27. Serine I. 28. Caprylic acid. 29. Leucine II. 30. Glycerol. 31. Phosphoric acid. 32. Threonine I. 33. Isoleucine II. 34. Proline II. 35. Glycine. 36. Glyceric acid. 37. Fumaric acid. 38. Serine II. 39. Pipecolic acid II. 40. Threonine II. 41. Aspartic acid I. 42. 3-Aminoisobutyric acid II. 43. Iminodiacetic acid I. 44. Threose II. 45. Aminomalonic acid. 46. Threitol. 47. Methionine II. 48. Pyroglutamic acid. 49. *trans*-4-Hydroxy-L-proline II. 50. Iminodiacetic acid II. 51. Phenylalanine I. 52. Creatinine. 53. Glutamic acid II. 54. Phenylalanine II. 55. Lauric acid. 56. Asparagine II. 57. Pyrophosphate. 58. Arabitol. 59. Xylitol. 60. Glutamine III. 61. Shikimic acid. 62. Hypoxanthine I. 63. OrnithineII. 64. Citric acid. 65. Myristic acid. 66. Galactopyranoside. 67. Pyranose (Glucose/Altrose/Galactose/Talose). 68. Furanose (Tagatose I). 69. *p*-Hydroxyphenyllactic acid. 70. Gluconic acid lactone I. 71. Tagatose II. 72. Fructose I. 73. Fructose II + Glucose. 74. Glucose. 75. Glucose. 76. Sorbitol. 77. Tyrosine II. 78. 3-Indoleacetic acid. 79. Galactopyranoside. 80. Palmitoleic acid. 81. Palmitic acid. 82. Uric acid I. 83. Methyl stearate (IS). 84. Tryptophan II. 85. Linoleic acid. 86. Oleic acid. 87. Arachidonic acid. 88. 1-Monomyristin. 89. Lactose II. 90. 5-Hydroxy-L-tryptophan I. 91. 2-Palmitoylglycerol. 92 1-Monopalmitin. 93. 2-Monostearin. 94. Glycerol monostearate. 95. Cholesterol.
(DOCX)

S2 Fig Comparison of LC-MS base peak chromatograms. Comparison of LC-MS base peak chromatograms for breast cancer (green) and control (blue). Panel A. Metabolic fingerprinting by LC-MS. B. Lipid fingerprinting by LC-MS. The marked windows are according to the compound class and their elution time.
(DOCX)

S3 Fig Comparison of ^1^H-NMR spectra. Comparison of ^1^H-NMR spectra for breast cancer (green) and control (blue). Chemical shifts of compounds with statistical significance are expanded.
(DOCX)

S4 Fig PCA score plots. PCA score plots for data set filtered by presence and reproducibility (green dots: breast cancer group; blue dots. control group; orange diamonds: quality control). Panel: A. MF by GC-MS. B. MF by NMR. C. MF by LC-MS(+)D. MF by LC-MS(-)E. LF by LC-MS(+). F. LF by LC-MS(-).
(DOCX)

S1 Table Number of features in each step of the data processing in the analytical methodologies used. (DOCX)

S2 Table Characteristic fragments of identified compounds identified by LC-MS/MS. (DOCX)

S3 Table Pathway analysis of metabolite alterations in breast cancer. (DOCX)
