# Supplementary material for: Multiplatform plasma metabolic and lipid fingerprinting of breast cancer: A pilot control-case study in Colombian Hispanic women
Source: PLoS One. 2018 Feb 13;13(2):e0190958. doi: 10.1371/journal.pone.0190958 (PMC5810980; doi:10.1371/journal.pone.0190958)
Supplement: S1 Fig — Comparison of GC-MS chromatograms (truncated at 30 minutes) for breast cancer (green) and control (blue). Identified metabolites: 1. N-Ethylglycine I. 2. Pyruvic acid. 3. Lactic acid. 4. Glycolic acid. 5. Valine I. 6. Alanine I. 7. 2-Ketoisocaproic acid I. 8. Acetoacetate I. 9. Glycine I. 10. 2-Hydroxybutyric acid I. 11. Sarcosine. 12. Acetoacetate II. 13. Oxalic acid. 14. p-Cresol. 15. Leucine I. 16. 3-Hydroxybutyric acid. 17. 2-etoisocaproic acid I. 18. N-methylalanine. 19. Proline I. 20. Isoleucine I. 21. 2-Ketoisocaproic acid II. 22. Malonic acid I. 23. Valine II. 24. Glyceraldehyde II. 25. Benzoic acid. 26. Urea. 27. Serine I. 28. Caprylic acid. 29. Leucine II. 30. Glycerol. 31. Phosphoric acid. 32. Threonine I. 33. Isoleucine II. 34. Proline II. 35. Glycine. 36. Glyceric acid. 37. Fumaric acid. 38. Serine II. 39. Pipecolic acid II. 40. Threonine II. 41. Aspartic acid I. 42. 3-Aminoisobutyric acid II. 43. Iminodiacetic acid I. 44. Threose II. 45. Aminomalonic acid. 46. Threitol. 47. Methionine II. 48. Pyroglutamic acid. 49. trans-4-Hydroxy-L-proline II. 50. Iminodiacetic acid II. 51. Phenylalanine I. 52. Creatinine. 53. Glutamic acid II. 54. Phenylalanine II. 55. Lauric acid. 56. Asparagine II. 57. Pyrophosphate. 58. Arabitol. 59. Xylitol. 60. Glutamine III. 61. Shikimic acid. 62. Hypoxanthine I. 63. OrnithineII. 64. Citric acid. 65. Myristic acid. 66. Galactopyranoside. 67. Pyranose (Glucose/Altrose/Galactose/Talose). 68. Furanose (Tagatose I). 69. p-Hydroxyphenyllactic acid. 70. Gluconic acid lactone I. 71. Tagatose II. 72. Fructose I. 73. Fructose II + Glucose. 74. Glucose. 75. Glucose. 76. Sorbitol. 77. Tyrosine II. 78. 3-Indoleacetic acid. 79. Galactopyranoside. 80. Palmitoleic acid. 81. Palmitic acid. 82. Uric acid I. 83. Methyl stearate (IS). 84. Tryptophan II. 85. Linoleic acid. 86. Oleic acid. 87. Arachidonic acid. 88. 1-Monomyristin. 89. Lactose II. 90. 5-Hydroxy-L-tryptophan I. 91. 2-Palmitoylglycerol. 92 1-Monopalmitin. 93. 2-Monostearin. 94. Glycer [file pone.0190958.s001.docx]

**Supporting Information**


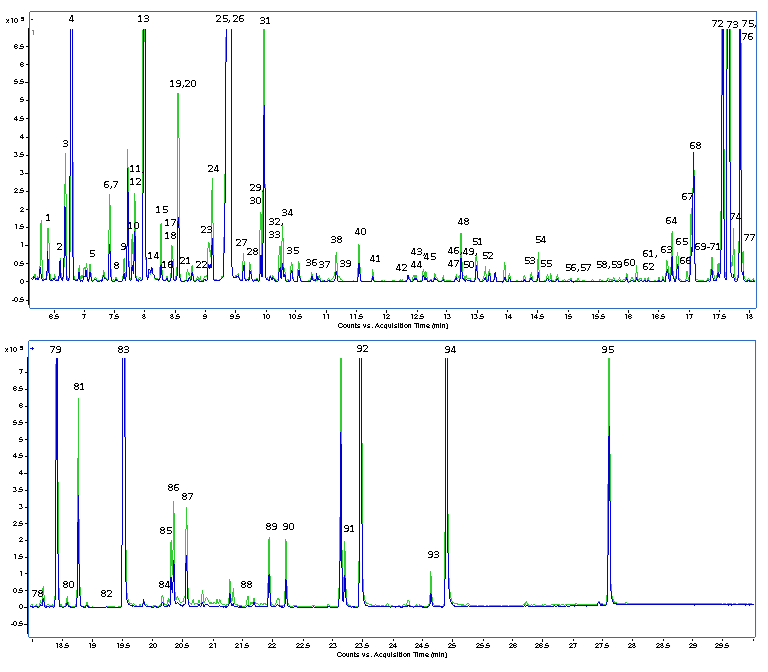


**S1 Fig. Comparison of GC-MS chromatograms.** Comparison of GC-MS chromatograms (truncated at 30 minutes) for breast cancer (green) and control (blue)**.** Identified metabolites: 1. *N*-Ethylglycine I. 2. Pyruvic acid. 3. Lactic acid. 4. Glycolic acid. 5. Valine I. 6. Alanine I. 7. 2-Ketoisocaproic acid I. 8. Acetoacetate I. 9. Glycine I. 10. 2-Hydroxybutyric acid I. 11. Sarcosine. 12. Acetoacetate II. 13. Oxalic acid. 14. *p*-Cresol. 15. Leucine I. 16. 3-Hydroxybutyric acid. 17. 2-etoisocaproic acid I. 18. N-methylalanine. 19. Proline I. 20. Isoleucine I. 21. 2-Ketoisocaproic acid II. 22. Malonic acid I. 23. Valine II. 24. Glyceraldehyde II. 25. Benzoic acid. 26. Urea. 27. Serine I. 28. Caprylic acid. 29. Leucine II. 30. Glycerol. 31. Phosphoric acid. 32. Threonine I. 33. Isoleucine II. 34. Proline II. 35. Glycine. 36. Glyceric acid. 37. Fumaric acid. 38. Serine II. 39. Pipecolic acid II. 40. Threonine II. 41. Aspartic acid I. 42. 3-Aminoisobutyric acid II. 43. Iminodiacetic acid I. 44. Threose II. 45. Aminomalonic acid. 46. Threitol. 47. Methionine II. 48. Pyroglutamic acid. 49. *trans*-4-Hydroxy-L-proline II. 50. Iminodiacetic acid II. 51. Phenylalanine I. 52. Creatinine. 53. Glutamic acid II. 54. Phenylalanine II. 55. Lauric acid. 56. Asparagine II. 57. Pyrophosphate. 58. Arabitol. 59. Xylitol. 60. Glutamine III. 61. Shikimic acid. 62.Hypoxanthine I. 63. OrnithineII. 64. Citric acid. 65. Myristic acid. 66. Galactopyranoside. 67. Pyranose (Glucose/Altrose/Galactose/Talose). 68. Furanose (Tagatose I). 69. *p*-Hydroxyphenyllactic acid. 70. Gluconic acid lactone I. 71. Tagatose II. 72. Fructose I. 73. Fructose II + Glucose. 74. Glucose. 75. Glucose. 76. Sorbitol. 77. Tyrosine II. 78. 3-Indoleacetic acid. 79. Galactopyranoside. 80. Palmitoleic acid. 81. Palmitic acid. 82. Uric acid I. 83. Methyl stearate (IS). 84. Tryptophan II. 85. Linoleic acid. 86. Oleic acid. 87. Arachidonic acid. 88. 1-Monomyristin. 89. Lactose II. 90. 5-Hydroxy-L-tryptophan I. 91. 2-Palmitoylglycerol. 92 1-Monopalmitin. 93. 2-Monostearin. 94. Glycerol monostearate. 95. Cholesterol.
