# Supplementary material for: Multiplatform plasma metabolic and lipid fingerprinting of breast cancer: A pilot control-case study in Colombian Hispanic women
Source: PLoS One. 2018 Feb 13;13(2):e0190958. doi: 10.1371/journal.pone.0190958 (PMC5810980; doi:10.1371/journal.pone.0190958)
Supplement: S2 Fig — Comparison of LC-MS base peak chromatograms for breast cancer (green) and control (blue). Panel A. Metabolic fingerprinting by LC-MS. B. Lipid fingerprinting by LC-MS. The marked windows are according to the compound class and their elution time. (DOCX) [file pone.0190958.s002.docx]

**Supporting Information**


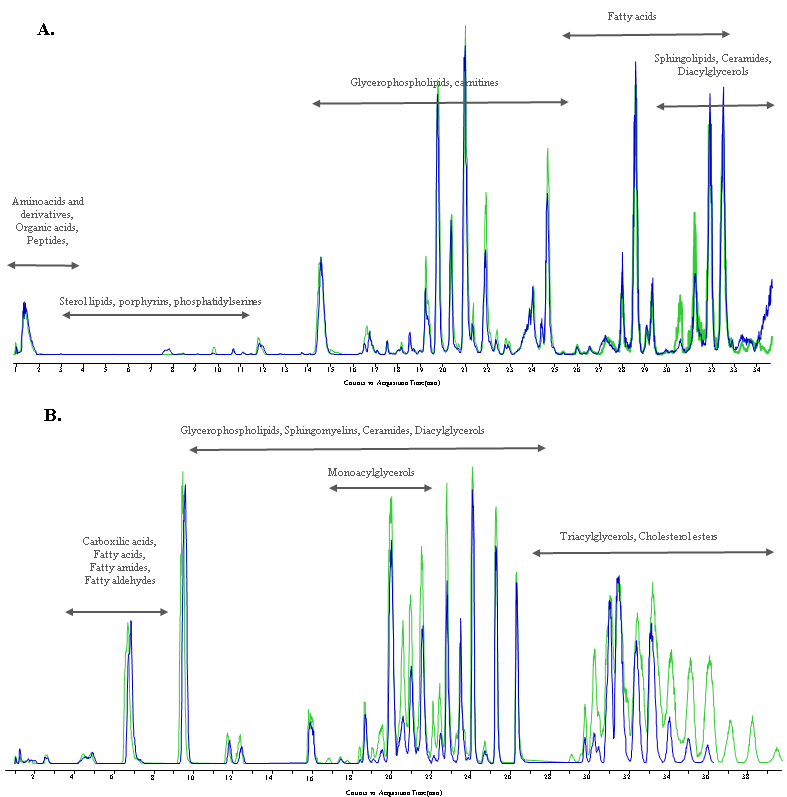


**S2 Fig. Comparison of LC-MS base peak chromatograms.** Comparison of LC-MS base peak chromatograms for breast cancer (green) and control (blue). Panel A. Metabolic fingerprinting by LC-MS. B. Lipid fingerprinting by LC-MS. The marked windows are according to the compound class and their elution time.
