# Supplementary material for: Multiplatform plasma metabolic and lipid fingerprinting of breast cancer: A pilot control-case study in Colombian Hispanic women
Source: PLoS One. 2018 Feb 13;13(2):e0190958. doi: 10.1371/journal.pone.0190958 (PMC5810980; doi:10.1371/journal.pone.0190958)
Supplement: S3 Fig — Comparison of 1H-NMR spectra for breast cancer (green) and control (blue). Chemical shifts of compounds with statistical significance are expanded. (DOCX) [file pone.0190958.s003.docx]

**Supporting Information**

**
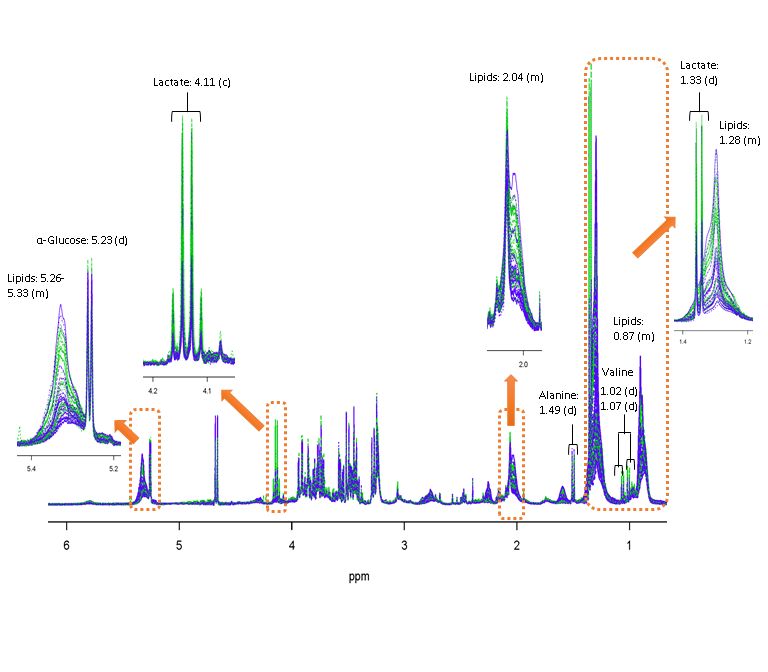
**

**S3 Fig. Comparison of ^1^H-NMR spectra.** Comparison of ^1^H-NMR spectra for breast cancer (green) and control (blue). Chemical shifts of compounds with statistical significance are expanded.
