# Supplementary material for: Multiplatform plasma metabolic and lipid fingerprinting of breast cancer: A pilot control-case study in Colombian Hispanic women
Source: PLoS One. 2018 Feb 13;13(2):e0190958. doi: 10.1371/journal.pone.0190958 (PMC5810980; doi:10.1371/journal.pone.0190958)
Supplement: S4 Fig — PCA score plots for data set filtered by presence and reproducibility (green dots: breast cancer group; blue dots. control group; orange diamonds: quality control). Panel: A. MF by GC-MS. B. MF by NMR. C. MF by LC-MS(+)D. MF by LC-MS(-)E. LF by LC-MS(+). F. LF by LC-MS(-). (DOCX) [file pone.0190958.s004.docx]

**Supporting Information**

**
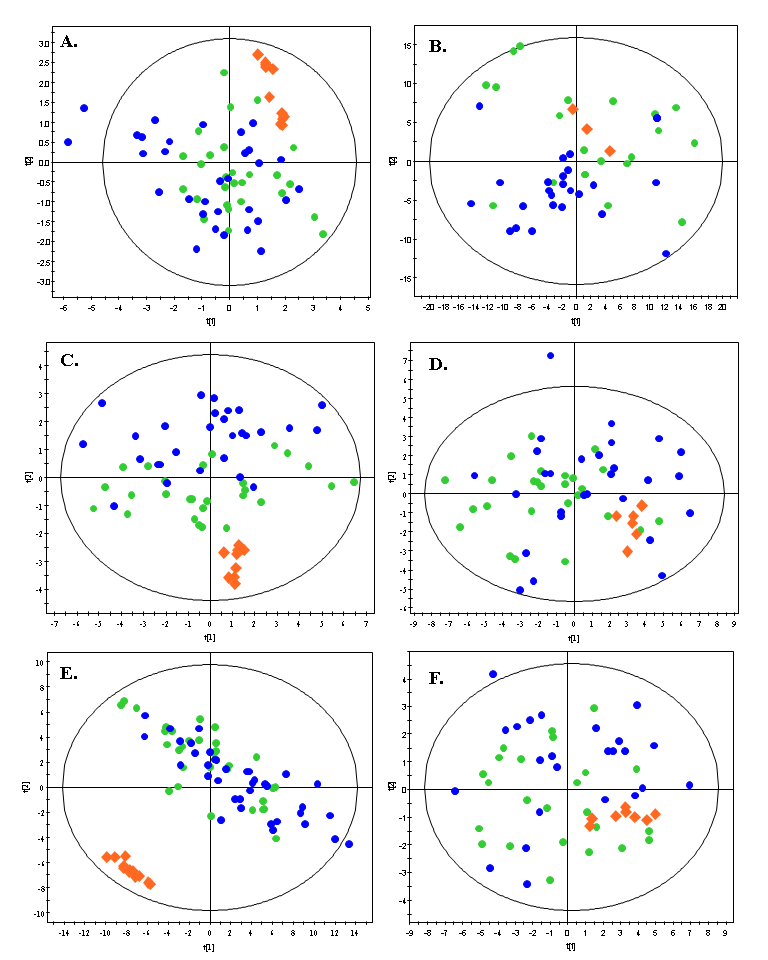
**

**S4 Fig.** **PCA score plots.** PCA score plots for data set filtered by presence and reproducibility (green dots: breast cancer group; blue dots. control group; orange diamonds: quality control). Panel: A. MF by GC-MS: *R^2^* =0.240. *Q^2^*=0.188. B. MF by NMR: *R^2^* =0.229. *Q^2^*=0.164. C. MF by LC-MS(+):*R^2^* =0.205. *Q^2^*=0.164. D. MF by LC-MS(-): *R^2^* =0.209. *Q^2^*=0.174. E. LF by LC-MS(+): *R^2^* =0.360. *Q^2^*=0.335. F. LF by LC-MS(-):*R^2^* =0.404. *Q^2^*=0.373.
