# Supplementary material for: Multiplatform plasma metabolic and lipid fingerprinting of breast cancer: A pilot control-case study in Colombian Hispanic women
Source: PLoS One. 2018 Feb 13;13(2):e0190958. doi: 10.1371/journal.pone.0190958 (PMC5810980; doi:10.1371/journal.pone.0190958)
Supplement: S1 Table — (DOCX) [file pone.0190958.s005.docx]

**Supporting Information**

**S1 Table.** Number of features in each step of the data processing in the analytical methodologies used.

|  |  | GC-MS | LC-MS | | | |
| --- | --- | --- | --- | --- | --- | --- |
|  |  |  | MF(+) | MF(-) | LF(+) | LF(-) |
| Number of features | After alignment | 120 | 810 | 961 | 1142 | 476 |
|  | After data treatment | 87 | 431 | 424 | 726 | 238 |
|  | After filtering by presence and CV | 77 | 298 | 313 | 532 | 238 |
|  | Statistically significant | 16 | 17 | 19 | 49 | 1 |
