# Supplementary material for: Multiplatform plasma metabolic and lipid fingerprinting of breast cancer: A pilot control-case study in Colombian Hispanic women
Source: PLoS One. 2018 Feb 13;13(2):e0190958. doi: 10.1371/journal.pone.0190958 (PMC5810980; doi:10.1371/journal.pone.0190958)
Supplement: S2 Table — (DOCX) [file pone.0190958.s006.docx]

**Supporting Information**

| **S2 Table.** Characteristic fragments of identified compounds identified by LC-MS/MS. | | | | | | | |
| --- | --- | --- | --- | --- | --- | --- | --- |
| **Compound name** | **Molecular Formula** | **Monoisotopic mass (Da)** | **RT** | **Mass error (ppm)** | **Analytical technique** | **DET** | **Fragments** |
| ***Fatty acyl carnitines*** |  |  |  |  |  |  |  |
| Decenoylcarnitine | C_17_H_31_NO_4_ | 313.2253 | 9.079 | 0 | MF by LC | ESI+ | 85.02924, 57.03518 |
| Decanoylcarnitine | C_17_H_33_NO_4_ | 315.2409 | 11.085 | 0 | MF by LC | ESI+ | 85.0288, 60.0815, 257.1715 |
| Dodecenoylcarnitine | C_19_H_35_NO_4_ | 341.2566 | 12.982 | 2 | MF by LC | ESI+ | 60.0834, 85.0305, 181.6594, 199.0148, 283.2703 |
| Tetradecadiencarnitine | C_21_H_37_NO_4_ | 367.2722 | 14.597 | 2 | MF by LC | ESI+ | 85.0280, 60.0914, 239.0476, 309.0538 |
| Lauroylcarnitine | C_19_H_37_NO_4_ | 343.2722 | 14.813 | 1 | MF by LC | ESI+ | 60.0838, 85.0291 |
| Tetradecenoylcarnitine | C_21_H_39_NO_4_ | 369.2879 | 16.351 | 0 | MF by LC | ESI+ | 60.0836, 85.0309, 144.1057, 311.2342 |
| Myristoylcarnitine | C_21_H_41_NO_4_ | 371.3035 | 18.065 | 0 | MF by LC | ESI+ | 85.0267 |
| Palmitoylcarnitine | C_23_H_45_NO_4_ | 399.3348 | 21.154 | 0 | MF by LC | ESI+ | 85.02911, 60.09296, 57.07618 |
| LPC(16:1) | C_24_H_48_NO_7_P | 493.3168 | 17.994 | 1 | MF by LC | ESI+ | 86.0965, 104.1071, 184.0728 |
| LPC(18:1) | C_26_H_52_NO_7_P | 521.3481 | 22.415 | 0 | MF by LC | ESI+ | 86.0971, 104.1076, 184.0735, 492.3465 |
| LPE(16:1) | C_21_H_42_NO_7_P | 451.2699 | 18.144 | 1 | MF by LC | ESI+ | 311.2572 |
| ***Fatty Acyls*** |  |  |  |  |  |  |  |
| Arachidonic acid | C_20_H_32_O_2_ | 304.24023 | 28.976 | 2 | MF by LC | ESI+ | 55.0558, 67.0546, 81.0698, 93.0697, 118.0871 |
|  |  |  |  |  |  | ESI- | 59.0139, 71.0176, 81.0386 |
| Linoleic acid | C_18_H_32_O_2_ | 280.24 | 29.349 | 2 | MF by LC | ESI+ | 69.0763, 83.0857, 97.1012, 109.1027 |
| Palmitic acid | C_16_H_32_O_2_ | 256.24 | 31.255 | 2 | MF by LC | ESI+ | 57.0716, 71.0873, 85.1020, 103.0791, 131.1048 |
| Oleic acid | C_18_H_34_O_2_ | 282.255 | 31.955 | 6 | MF by LC | ESI+ | 57.0709, 69.0706, 81.0705, 93.0692 |
| ***Phosphatidylcholines*** |  |  |  |  |  |  |  |
| PC(32:1) | C_40_H_78_NO_8_P | 731.5465 | 20.68 | 0 | LF by LC | ESI+ | 184.0733 |
| PC(36:0) | C_44_H_88_NO_8_P | 789.6248 | 21.81 | 6 | LF by LC | ESI+ |  |
| PC(36:1) | C_44_H_86_NO_8_P | 787.6091 | 21.79 | 0 | LF by LC | ESI+ | 86.0966, 184.0730 |
| PC(38:4) | C_46_H_84_NO_8_P | 809.5935 | 21.81 | 10 | LF by LC | ESI+ | 86.0998,184.0752 |
| PC(38:6) | C_46_H_80_NO_8_P | 805.5622 | 19.52 | 0 | LF by LC | ESI+ | 86.0973,184.0721, 653.5026 |
| PC(38:7) | C_46_H_78_NO_8_P | 803.5465 | 19.54 | 0 | LF by LC | ESI+ | 124.9988, 184.0728 |
| PC(40:5) | C_48_H_86_NO_8_P | 835.6091 | 21.98 | 0 | LF by LC | ESI+ | 184.0750 |
| PC(40:6) | C_48_H_84_NO_8_P | 833.5935 | 21.12 | 0 | LF by LC | ESI+ | 86.1001, 184.0739 |
| PC(O-36:4) | C_44_H_82_NO_7_P | 765.5672 | 21.33 | 1 | LF by LC | ESI+ | 86.0945, 184.0729 |
| PC(P-38:4) | C_46_H_84_NO_7_P | 793.5985 | 22.13 | 2 | LF by LC | ESI+ | 86.0966, 184.0724 |
| ***Sphingolipid*** |  |  |  |  |  |  |  |
| SM(d41:2) | C_46_H_91_N_2_O_6_P | 798.6615 | 22.74 | 1 | LF by LC | ESI+ | 184.0727 |
| SM(d42:2) | C_47_H_93_N_2_O_6_P | 812.6771 | 23.34 | 0 | LF by LC | ESI+ | 184.0725 |
| ***Triacylglycerides*** |  |  |  |  |  |  |  |
| TG(48:0) | C_51_H_98_O_6_ | 806.7363 | 31.7 | 1 | LF by LC | ESI+ | 549.4873, 575.5031, 601.5171 |
| TG(48:1) | C_51_H_96_O_6_ | 804.7207 | 30.38 | 3 | LF by LC | ESI+ | 521.4605, 545.4537, 575.5018 |
| TG(48:3) | C_51_H_92_O_6_ | 800.6894 | 29.89 | 1 | LF by LC | ESI+ | 523.4660, 549.9797, 577.5116 |
| TG(50:0) | C_53_H_102_O_6_ | 834.7676 | 33.98 | 5 | LF by LC | ESI+ | 565.51553, 575.4940, 589.5224 |
| TG(50:2) | C_53_H_98_O_6_ | 830.7363 | 31.01 | 1 | LF by LC | ESI+ | 561.4961, 575.5085, 589.5024 |
| TG(50:3) | C_53_H_96_O_6_ | 828.7207 | 31.69 | 1 | LF by LC | ESI+ | 549.4834, 571.4744, 595.4705 |
| TG(50:4) | C_53_H_94_O_6_ | 844.7389 | 30.38 | 3 | LF by LC | ESI+ | 547.4712, 569.4491, 601.5158 |
| TG(52:0) | C_55_H_106_O_6_ | 862.7989 | 37.05 | 2 | LF by LC | ESI+ | 575.5024, 603.5338, 631.5657 |
| TG(52:1) | C_55_H_104_O_6_ | 860.7833 | 34.89 | 5 | LF by LC | ESI+ | 577.5197, 603.5344, 627.5346 |
| TG(52:2) | C_55_H_102_O_6_ | 858.7676 | 34 | 10 | LF by LC | ESI+ | 577.5170, 601.5178 |
| TG(52:3) | C_55_H_100_O_6_ | 856.7520 | 32.36 | 9 | LF by LC | ESI+ | 577. 5192, 599.5028, 626.5178 |
| TG(52:4) | C_55_H_98_O_6_ | 854.7363 | 32.36 | 1 | LF by LC | ESI+ | 563.4992, 587.5016, 599.4981 |
| TG(52:5) | C_55_H_96_O_6_ | 852.7207 | 30.99 | 0 | LF by LC | ESI+ | 575.5079, 595.4648, 619.4665 |
| TG(54:2) | C_57_H_106_O_6_ | 886.7989 | 39.43 | 1 | LF by LC | ESI+ | 603.4715, 44.9297, 677.9635 |
| TG(54:3) | C_57_H_104_O_6_ | 884.7833 | 37.07 | 1 | LF by LC | ESI+ | 603.5282, 626.5288, 652.5386 |
| TG(54:4) | C_57_H_102_O_6_ | 882.7676 | 34.9 | 1 | LF by LC | ESI+ | 603.5324, 623.5052 |
| TG(54:6) | C_57_H_98_O_6_ | 878.7363 | 31.59 | 2 | LF by LC | ESI+ | 573.5968, 599.5094, 621.4861 |
| TG(56:5) | C_59_H_104_O_6_ | 908.7833 | 36.1 | 0 | LF by LC | ESI+ | 530.2568, 591.5390, 655.8471 |
| TG(56:6) | C_59_H_102_O_6_ | 906.7676 | 34.79 | 0 | LF by LC | ESI+ | 603.5329, 626.5013, 647.5008 |
| TG(56:7) | C_59_H_100_O_6_ | 904.7520 | 33.3 | 0 | LF by LC | ESI+ | 577.5219, 623.4781, 649.5339 |
| TG(56:8) | C_59_H_98_O_6_ | 902.7363 | 31.73 | 3 | LF by LC | ESI+ | 597.5072, 623.4864, 645.4927 |
