# Supplementary material for: Multiplatform plasma metabolic and lipid fingerprinting of breast cancer: A pilot control-case study in Colombian Hispanic women
Source: PLoS One. 2018 Feb 13;13(2):e0190958. doi: 10.1371/journal.pone.0190958 (PMC5810980; doi:10.1371/journal.pone.0190958)
Supplement: S3 Table — (DOCX) [file pone.0190958.s007.docx]

**Supporting Information**

**S3 Table.** Pathway analysis of metabolite alterations in breast cancer.

| Pathway name | Total | Expected | Hits | Raw p | -Log(p) | Holm adjust | FDR | Impact |
| --- | --- | --- | --- | --- | --- | --- | --- | --- |
| Aminoacyl-tRNA biosynthesis | 75 | 0.90361 | 5 | 0.001699 | 6.3777 | 0.13592 | 0.071631 | 0.11268 |
| Fatty acid biosynthesis | 49 | 0.59036 | 4 | 0.0024768 | 6.0008 | 0.19566 | 0.071631 | 0 |
| Alanine, aspartate and glutamate metabolism | 24 | 0.28916 | 3 | 0.0026862 | 5.9196 | 0.20952 | 0.071631 | 0.23362 |
| Valine, leucine and isoleucine biosynthesis | 27 | 0.3253 | 3 | 0.0037887 | 5.5757 | 0.29173 | 0.075774 | 0.04823 |
| Glycerolipid metabolism | 32 | 0.38554 | 3 | 0.0061697 | 5.0881 | 0.4689 | 0.098716 | 0.25239 |
| Propanoate metabolism | 35 | 0.42169 | 3 | 0.0079462 | 4.8351 | 0.59597 | 0.10595 | 0 |
| Glycerophospholipid metabolism | 39 | 0.46988 | 3 | 0.010743 | 4.5335 | 0.79496 | 0.12277 | 0.20482 |
| Linoleic acid metabolism | 15 | 0.18072 | 2 | 0.013357 | 4.3157 | 0.97509 | 0.13357 | 0.65625 |
| Taurine and hypotaurine metabolism | 20 | 0.24096 | 2 | 0.023288 | 3.7598 | 1 | 0.20701 | 0.05395 |
| Cysteine and methionine metabolism | 56 | 0.6747 | 3 | 0.028415 | 3.5608 | 1 | 0.22732 | 0.02846 |
| Pantothenate and CoA biosynthesis | 27 | 0.3253 | 2 | 0.040847 | 3.1979 | 1 | 0.29707 | 0 |
| Glycolysis or Gluconeogenesis | 31 | 0.37349 | 2 | 0.05254 | 2.9462 | 1 | 0.34235 | 0.0953 |
| Pyruvate metabolism | 32 | 0.38554 | 2 | 0.055632 | 2.889 | 1 | 0.34235 | 0.3201 |
| Arginine and proline metabolism | 77 | 0.92771 | 3 | 0.063379 | 2.7586 | 1 | 0.36216 | 0.1068 |
| Valine, leucine and isoleucine degradation | 40 | 0.48193 | 2 | 0.082494 | 2.495 | 1 | 0.41247 | 0 |
| Butanoate metabolism | 40 | 0.48193 | 2 | 0.082494 | 2.495 | 1 | 0.41247 | 0.08516 |
| Glycine, serine and threonine metabolism | 48 | 0.57831 | 2 | 0.11253 | 2.1845 | 1 | 0.52662 | 0.13604 |
| Fatty acid metabolism | 50 | 0.60241 | 2 | 0.12045 | 2.1165 | 1 | 0.52662 | 0.02959 |
| D-Glutamine and D-glutamate metabolism | 11 | 0.13253 | 1 | 0.12507 | 2.0789 | 1 | 0.52662 | 0.1123 |
| Arachidonic acid metabolism | 62 | 0.74699 | 2 | 0.17048 | 1.7691 | 1 | 0.67351 | 0.21669 |
| Cyanoamino acid metabolism | 16 | 0.19277 | 1 | 0.1768 | 1.7328 | 1 | 0.67351 | 0 |
| Sulfur metabolism | 18 | 0.21687 | 1 | 0.19665 | 1.6263 | 1 | 0.71509 | 0 |
| Citrate cycle (TCA cycle) | 20 | 0.24096 | 1 | 0.21604 | 1.5323 | 1 | 0.75144 | 0.09024 |
| Selenoamino acid metabolism | 22 | 0.26506 | 1 | 0.23498 | 1.4483 | 1 | 0.78325 | 0 |
| Sphingolipid metabolism | 25 | 0.3012 | 1 | 0.26256 | 1.3373 | 1 | 0.84018 | 0 |
| Fatty acid elongation in mitochondria | 27 | 0.3253 | 1 | 0.28041 | 1.2715 | 1 | 0.84914 | 0 |
| beta-Alanine metabolism | 28 | 0.33735 | 1 | 0.28917 | 1.2407 | 1 | 0.84914 | 0.01119 |
| alpha-Linolenic acid metabolism | 29 | 0.3494 | 1 | 0.29784 | 1.2112 | 1 | 0.84914 | 0 |
| Pentose phosphate pathway | 32 | 0.38554 | 1 | 0.32323 | 1.1294 | 1 | 0.84914 | 0 |
| Vitamin B6 metabolism | 32 | 0.38554 | 1 | 0.32323 | 1.1294 | 1 | 0.84914 | 0.01914 |
| Terpenoid backbone biosynthesis | 33 | 0.39759 | 1 | 0.33149 | 1.1042 | 1 | 0.84914 | 0 |
| Methane metabolism | 34 | 0.40964 | 1 | 0.33966 | 1.0798 | 1 | 0.84914 | 0.01751 |
| Glutathione metabolism | 38 | 0.45783 | 1 | 0.37137 | 0.99056 | 1 | 0.86843 | 0.01095 |
| Nitrogen metabolism | 39 | 0.46988 | 1 | 0.37906 | 0.97005 | 1 | 0.86843 | 0 |
| Galactose metabolism | 41 | 0.49398 | 1 | 0.39418 | 0.93094 | 1 | 0.86843 | 0 |
| Nicotinate and nicotinamide metabolism | 44 | 0.53012 | 1 | 0.4162 | 0.8766 | 1 | 0.86843 | 0 |
| Histidine metabolism | 44 | 0.53012 | 1 | 0.4162 | 0.8766 | 1 | 0.86843 | 0.00051 |
| Phenylalanine metabolism | 45 | 0.54217 | 1 | 0.42336 | 0.85953 | 1 | 0.86843 | 0 |
| Ascorbate and aldarate metabolism | 45 | 0.54217 | 1 | 0.42336 | 0.85953 | 1 | 0.86843 | 0.01617 |
| Lysine degradation | 47 | 0.56627 | 1 | 0.43744 | 0.82682 | 1 | 0.87487 | 5.00E-04 |
| Glyoxylate and dicarboxylate metabolism | 50 | 0.60241 | 1 | 0.45793 | 0.78104 | 1 | 0.89352 | 0 |
| Pentose and glucuronate interconversions | 53 | 0.63855 | 1 | 0.4777 | 0.73877 | 1 | 0.90991 | 0 |
| Pyrimidine metabolism | 60 | 0.72289 | 1 | 0.52117 | 0.65169 | 1 | 0.96961 | 0 |
| Tyrosine metabolism | 76 | 0.91566 | 1 | 0.6078 | 0.49792 | 1 | 1 | 0 |
| Porphyrin and chlorophyll metabolism | 104 | 1.253 | 1 | 0.72433 | 0.3225 | 1 | 1 | 0 |
